# Financial reimbursement for clinical trial participation costs: a pilot feasibility study

**DOI:** 10.1093/jncics/pkag048

**Published:** 2026-05-03

**Authors:** Courtney P Williams, Nicole L Henderson, Nusrat Jahan, Erica Stringer-Reasor, Andres Azuero, Maria Pisu, Rebecca C Arend, Gabrielle B Rocque

**Affiliations:** Division of General Internal Medicine & Population Science, University of Alabama at Birmingham, Birmingham, AL, United States; O’Neal Comprehensive Cancer Center, University of Alabama at Birmingham, Birmingham, AL, United States; O’Neal Comprehensive Cancer Center, University of Alabama at Birmingham, Birmingham, AL, United States; Division of Hematology & Oncology, University of Alabama at Birmingham, Birmingham, AL, United States; Division of Hematology & Oncology, University of Alabama at Birmingham, Birmingham, AL, United States; Division of Hematology & Oncology, University of Alabama at Birmingham, Birmingham, AL, United States; School of Nursing, University of Alabama at Birmingham, Birmingham, AL, United States; Division of General Internal Medicine & Population Science, University of Alabama at Birmingham, Birmingham, AL, United States; O’Neal Comprehensive Cancer Center, University of Alabama at Birmingham, Birmingham, AL, United States; O’Neal Comprehensive Cancer Center, University of Alabama at Birmingham, Birmingham, AL, United States; Department of Obstetrics and Gynecology, University of Alabama at Birmingham, Birmingham, AL, United States; O’Neal Comprehensive Cancer Center, University of Alabama at Birmingham, Birmingham, AL, United States; Division of Hematology & Oncology, University of Alabama at Birmingham, Birmingham, AL, United States

## Abstract

**Background:**

Clinical trial participants rarely represent the real-world treatment population, potentially due to costs associated with participation. Monetary reimbursement for trial-related costs could address financial barriers to trial recruitment and retention.

**Methods:**

This mixed-methods, pilot, feasibility study provided financial reimbursement to women with breast cancer participating in a clinical trial. Patients were reimbursed $1000/month during their first 4 months of trial participation, surveyed biweekly to assess changes in financial toxicity, and then interviewed to explore the effects of receiving reimbursement on trial-related costs and recruitment and retention. Mixed-effect modeling and thematic analysis were completed. Feasibility was defined as 80% retention of patients on the reimbursement study, with those retained completing 75% of surveys.

**Results:**

Of 39 consented patients, 33 patients completed the pilot study (85% retention, 100% survey completion). Patients were a median 52 years old (interquartile range = 44-59), 48% Black, 67% privately insured, and 42% found it difficult to live on their current income. Patient financial toxicity modestly decreased. Patients (n = 32) reported using the reimbursement to pay for trial visit-related food, transportation, caregiver expenses, and out-of-pocket medical costs. Patients felt receiving reimbursement affected trial retention more so than recruitment, stating “I would have enrolled regardless … [but knowing] it wasn’t going to place a financial strain on us because of these reimbursements. … It made it easier for me to feel good about continuing.”

**Conclusion:**

Reimbursement for clinical trial-related costs is feasible, suggests decreases in financial toxicity, and is a promising approach to improve trial retention outcomes in women with breast cancer.

**ClinicalTrials.gov ID:**

NCT05871125

## Background

Although cancer clinical trials are crucial to advancing the science of cancer treatment and improving survival for patients, 1 in 4 cancer clinical trials do not attain complete accrual, and 1 in 5 close with <50% target accrual after 3 years.^1^^,^^2^ Furthermore, it is estimated that 12%-26% of patients with cancer who enroll in a therapeutic clinical trial drop out, withdraw their consent, or are lost to follow-up before study completion.^3-6^ Disparities in trial participation also exist, with minoritized patients having worse accrual and retention rates than their White counterparts.^7-9^ Issues surrounding cancer clinical trial participation are concerning and can result in selection bias, loss of validity due to decreases in sample size and statistical power, and a reduction in the pace of research progress due to prolonged trial durations. A commonly cited cause of barriers to clinical trial accrual and retention are patient-related burdens required for participation, including those related to out-of-pocket costs.^10^^,^^11^

Financial hardship is a commonly recognized side effect of standard-of-care cancer diagnosis and treatment.^12-14^ However, it is also experienced by oncology clinical trial populations. Because insurers only cover standard-of-care expenses, patients are responsible for research costs not covered by the trial sponsor, such as trial-related labs, imaging, or out-of-network care, as well as indirect costs such as travel and time off work for frequent clinical visits.^15-18^ Thus, nearly half of patients in early-phase cancer clinical trials report spending >$1000 per month out-of-pocket to participate, and 47% of cancer clinical trial participants report financial hardship as a result of participation.^19^^,^^20^ Furthermore, patients with lower incomes have 32% lower odds of clinical trial participation compared with those with higher incomes.^21^ Therefore, strategies addressing trial-related financial burden are urgently needed due to their potential to increase cancer clinical trial participation rates.

Financial reimbursement for trial-related expenses represents a potential strategy to increase trial participation rates by reducing trial-related financial hardship.^22^ Our previous nationwide survey found 1 in 4 respondents reported that payment or support for trial participation, such as transportation, child care, or paid time off from work, would influence their decision to participate,^22^ and another study of cancer survivors found this number to be closer to 80%.^23^ Other studies have found increases in clinical trial enrollment and decreases in financial hardship for patients receiving reimbursement for trial expenses at institutions in the US Northeast and West Coast regions.^24^^,^^25^ However, the impact of reimbursement on clinical trial participation, including recruitment and retention, for patients receiving cancer care in the US Deep South, where disparate barriers to accessing care exist compared with other areas of the country, is currently unclear. Additionally, criticisms surrounding the ethics of reimbursement exist, such as coercion or undue influence on participation, equity considerations regarding differential payment amounts, and regulatory uncertainties.^26-29^ However, the patient perspective on reimbursement for clinical trial expenses is less understood. Therefore, this study explores the feasibility and preliminary impact of a pilot financial reimbursement intervention for women with breast cancer living in the Deep South who are eligible for a clinical trial.

## Methods

### Study design and population

This convergent, mixed-methods, pilot, feasibility, single-arm trial provided financial reimbursement to therapeutic clinical trial-eligible women with breast cancer at the University of Alabama at Birmingham from July 2023 to June 2025. The study sample included English-speaking women with early-stage (II-III) or metastatic (de novo or recurrent) breast cancer either (1) considered eligible for and currently enrolling in a clinical trial, or (2) within 30 days of enrollment in a clinical trial at the UAB Medical Oncology Clinic who were at risk for or experiencing financial toxicity. Financial toxicity was identified in 1 of 2 ways. First, through oncologist or clinical trial nurse referrals based on known patient social needs, such as living far away from UAB or having small children at home. Second, through standard-of-care, patient-reported financial toxicity data collected in clinic (COmprehensive Score for financial Toxicity^30^ [COST]; scored 0-44, lower scores indicate worse financial toxicity; scores <26 were eligible, consistent with established thresholds for moderate-to-severe financial toxicity^31-33^). Patients with identified social needs did not need to meet the COST score criteria for study eligibility. Eligible patients were informed about the reimbursement study by their oncologist or clinical trial nurse, approached during their clinic visit, and, if interested, provided with an informed consent form by a study coordinator. This study was approved by the UAB Institutional Review Board (IRB-300009554; ClinicalTrials.gov ID: NCT05871125).

### Intervention: Financial reimbursement

We used a dose de-escalation design to assess the effects of financial reimbursement for clinical trial participation,^34^^,^^35^ assuming financial toxicity may increase when the dollar amount of the financial reimbursement “dose” decreases. This is a novel health services research methodology modeled after a phase I dose de-escalation clinical trial design to find the minimal effective “dose” of monetary reimbursement needed to combat clinical trial-related financial toxicity.^36^^,^^37^ Patients were “dosed” in cohorts of 5, with the first cohort receiving a financial reimbursement of $1000 per month for their first 4 months via a Greenphire ClinCard, for a total of $4000 received per patient (Figure S1). The $1000-per-month reimbursement rate and de-escalation approach was determined based on results from 3 studies completed in other US regions, as well as wanting to provide adequate compensation for the unique cost-related barriers to trial participation in Alabama-based patients.^19^^,^^38^^,^^39^ During those 4 months, patients were surveyed biweekly to determine their financial toxicity (COST), financial reimbursement acceptability (Acceptability of Intervention Measure [AIM]^40^), and financial reimbursement appropriateness (Intervention Appropriateness Measure [IAM]^40^). At the end of the 4-month period, suitability of the financial reimbursement dollar amount was determined. The reimbursement amount was considered suitable if at least 4 of 5 patients had a negative financial toxicity screen (average COST ≥26) and found the financial reimbursement dose acceptable and appropriate (average AIM and IAM score ≥4; Table S1). The reimbursement amount was found unsuitable if at least 1 of the 3 suitability criteria was not met in 2 or more patients, reflecting a “target toxicity level” of 40%.^36^ If the reimbursement dose was found suitable, the dollar amount of the financial reimbursement was de-escalated for the next cohort of 5 patients. If the dollar amount of the financial reimbursement was found unsuitable, the next cohort of 5 patients received the same reimbursement amount ($1000 per month for 4 months).

### Primary outcome: Feasibility

Feasibility benchmarks were defined as 80% retention of consented patients in the reimbursement study and retained patients completing at least 75% of biweekly surveys while enrolled. Feasibility was also assessed by the patient-reported acceptability (as measured by the AIM; scored 1-5, higher scores indicate greater acceptability) and appropriateness (as measured by the IAM; scored 1-5, higher scores indicate greater appropriateness) of the financial reimbursement.

### Secondary quantitative outcomes: Financial hardship

Secondary outcomes captured using biweekly surveys emailed to patients via RedCAP^41^^,^^42^ included financial toxicity (as measured by the COST), material financial hardship (13 domains, including trouble paying for medical expenses or basic needs), and behavioral financial hardship (12 domains, including postponing or skipping recommended care). Patients unable to complete surveys digitally were called by the study coordinator to complete the survey. Trajectories of these measures were assessed over the 4-month time frame while patients were actively receiving financial reimbursement.

### Secondary qualitative outcomes: Impact on trial recruitment, retention, and financial hardship

After receiving their last financial reimbursement, patients were asked to engage in a video-call-based semistructured interview to explore effects of the financial reimbursement on trial recruitment, retention, and financial hardship. Interviews were conducted by a health services researcher (CW) using interview guides (Figure S2) developed in conjunction with a medical anthropologist (NH), breast cancer medical oncologist (GR), health economist (MP), and patient advocate. Interviews were recorded and digitally transcribed (rev.com).

### Variables

Baseline surveys collected patient sociodemographic data, including highest level of education completed, employment status, marital status, and feelings about household income. Other patient sociodemographic data (date of birth, race, address, insurance status), cancer characteristics (diagnosis date, stage, previous treatments), and clinical trial data (trial enrollment date, first trial treatment date) were abstracted from the electronic medical record. Patient address was used to calculate residence rurality using Rural-Urban Continuum Codes.^43^ All quantitative data were collected and stored via RedCAP.^41^^,^^42^

### Sample size

Because this was a pilot study to assess feasibility of methods to provide trial-related cost reimbursement to patients in a larger-scale study, no true power and sample size calculation was applicable for the quantitative analyses.^44^^,^^45^ For qualitative analyses, the sample size was sufficient for meeting theoretical saturation and appropriate for this study.^46^

### Statistical analysis

*Quantitative data:* Descriptive sociodemographic, survey, and feasibility data were calculated for all patients at baseline and over the course of the study. Mean differences, or effect sizes, were calculated using Cohen’s d or Cramer’s V to determine the magnitude of relationships in bivariate associations.^47^ Associations between the financial reimbursement (1) acceptability, (2) appropriateness, and (3) patient-reported financial hardship and study time were estimated using fixed-effect beta coefficients (β), estimated marginal means (x̄), and corresponding 95% confidence intervals (CIs) from mixed-effect models. Comparisons by race (White/Black) were investigated. Analyses were conducted in SAS 9.4 software.

*Qualitative data*: Transcribed interviews were coded using a framework approach by a medical anthropologist (NH), who first familiarized herself with the interviews. Next, she coded thematically, organized the codes into an analytical framework, applied the analytical framework, and then charted the data into a framework matrix for interpretation. To assess thematic concordance, second coding was completed by a research assistant (HR) with strong intercoder reliability (pooled k = 0.85). Using iterative content analysis, prominent themes, patterns, and quotes were identified from the transcripts. All analyses were completed using DeDoose 9.0.107.^48^

## Results

### Study sample and feasibility results

**Figure 1.**
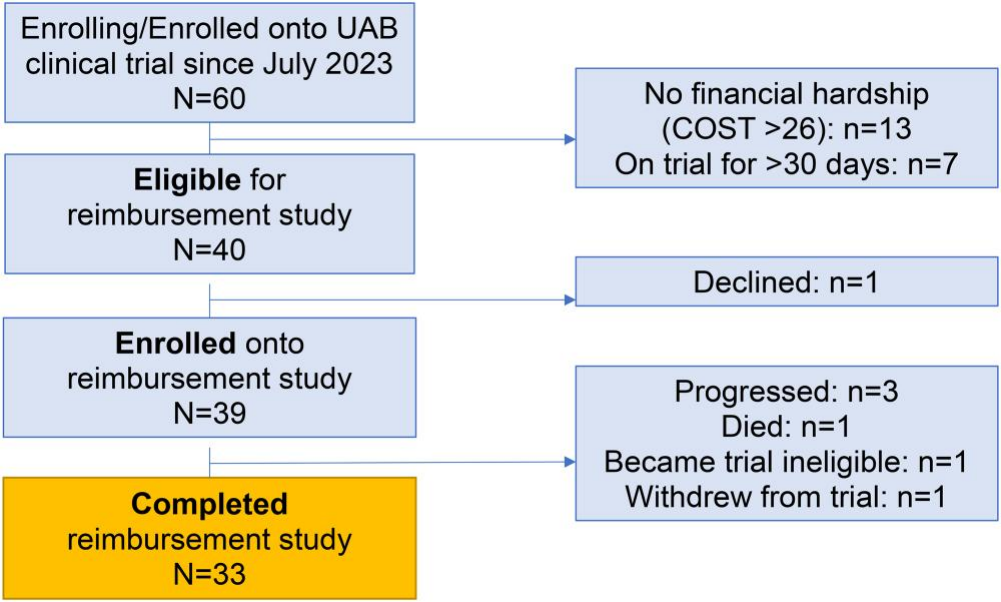
Consort diagram.

**Figure 2.**
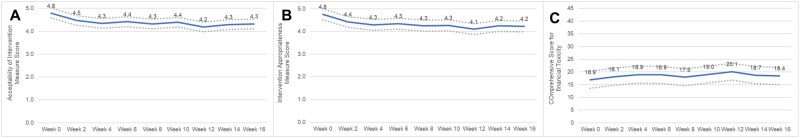
Mixed-model estimates of change over time for the (**A**) Acceptability of Intervention Measure Score, (**B**) Intervention Appropriateness Measure Score, and (C) COmprehensive Score for financial Toxicity (N = 33).

Of 60 women enrolled into a UAB breast cancer clinical trial during our study time frame, 40 (67%) were eligible for this reimbursement study. Of those eligible, 39 enrolled and 33 completed this study, including 21 women with early-stage and 12 women with metastatic breast cancer (Figure 1). This translates to an 85% retention rate with 100% survey completion, surpassing our feasibility benchmarks. Notably, no patient dropped out of the reimbursement study; all attrition was due to becoming ineligible for the parent clinical trial or death. Patients in our study were a median 52 years old (interquartile range = 44-59), 48% were Black or a person of color, 39% had a college degree, 58% were employed, 67% were privately insured, and 42% reported finding it difficult to live on their household income (Table 1). Over the course of the study, patients reported the financial reimbursement to be highly acceptable (lowest x̄ = 4.2 [95% CI = 4.0 to 4.4]; Figure 2A) and appropriate (lowest x̄ = 4.1 [95% CI = 3.9 to 4.3]; Figure 2B).

**Table 1.** Patient sociodemographic and clinical characteristics (N = 33).

	n (%)
Age at trial enrollment (median, IQR)	52 (44-59)
Race	
White	17 (52%)
Black	16 (48%)
Education	
High school or less	9 (27%)
Some college	11 (33%)
College degree	13 (39%)
Occupational status	
Employed	19 (58%)
Unemployed/disabled	7 (21%)
Other	7 (21%)
Marital status	
Married or living as married	14 (42%)
Divorced/widowed/separated	11 (33%)
Single, never married	8 (24%)
Residence	
Rural	5 (15%)
Urban or suburban	28 (85%)
Feelings about household income	
Living comfortably or getting by	19 (58%)
Finding it difficult or very difficult	14 (42%)
Health insurance status	
Private	22 (67%)
Medicare/Medicaid	11 (33%)
Breast cancer stage	
II/III	21 (64%)
IV/Recurrent	12 (36%)
Days since breast cancer diagnosis (median, IQR)	69 (45-349)
Previous systemic treatment	27 (82%)
Previous surgical treatment	13 (39%)
Previous radiation therapy	7 (21%)

Abbreviation: IQR = interquartile range.

### Financial hardship

**Figure 3.**
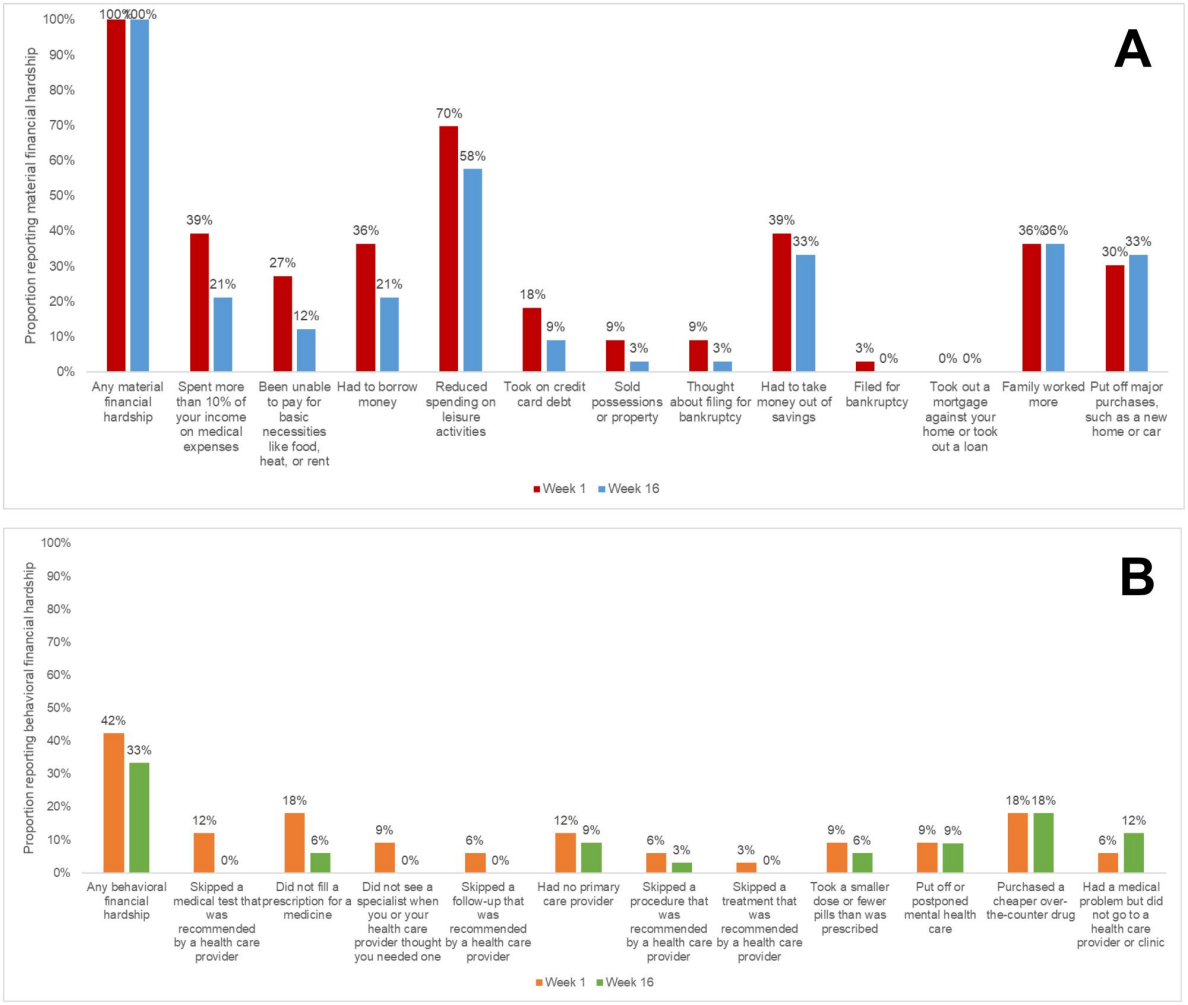
Proportions of patients reporting (**A**) material or (**B**) behavioral financial hardship at start and end of study (N = 33).

No patient cohort met the criteria for reimbursement de-escalation. Thus, the financial reimbursement amount was kept at $1000 per month for all participants. Over the course of the study, patient financial toxicity stayed high but modestly decreased (COST β = 1.5, 95% CI = −0.5 to 3.5; Figure 2C). This effect differed by race, with Black patients having greater financial toxicity at the start of the study (Black Week 1 COST x̅ = 16.6, 95% CI = 12.6 to 20.7; White Week 1 COST x̅ = 19.5, 95% CI = 14.0 to 25.0) but also greater decreases compared with White patients (Black COST β = 2.3, 95% CI = −0.7 to 5.2; White COST β = 0.8, 95% CI = −1.9 to 3.4). All patients reported at least 1 domain of material financial hardship at every time point in our study. However, decreases in material financial hardship were reported over time, including having to spend >10% of income on medical expenses (Week 1: 39% vs Week 16: 21%), having to borrow money (Week 1: 36% vs Week 16: 21%), and being unable to pay for basic necessities like food, heat, or rent (Week 1: 27% vs Week 16: 12%; Figure 3A). At least 1 domain of behavioral financial hardship was reported by 18%-42% of patients over the course of our study, with decreases seen in adherence measures, including skipping a recommended medical test (Week 1: 12% vs Week 16: 0%), due specialist (Week 1: 9% vs Week 16: 0%), or follow-up appointment (Week 1: 6% vs Week 16: 0%; Figure 3B).

### Qualitative results

Qualitative interviews (N = 32) revealed patient perspectives regarding (1) the trial-related expenses incurred, the impact of reimbursement on (2) trial recruitment and retention and (3) financial hardship, and (4) suggested modifications to the financial reimbursement intervention (Table 2).

**Table 2.** Qualitative themes, subthemes, and pertinent quotes (N = 33).

Theme	Subtheme	PID	Pertinent quote
Trial-related expenses incurred	Food	25	I had to change my diet. I couldn’t eat how I was eating. I had to eat things that were healthier. The study that I’m on now, lots of diarrhea, lots of nausea, lots of it. And so I had to change my diet from the way I eat to more vegetables. Of course, eating healthy costs.
	Transportation	8	[I used the money for] maintaining my vehicle because that is a lot of driving, getting my oil changes and stuff, trying to take care of my car even though it sounds like it want to fall apart.
	Caregiving	12	I used [the reimbursement] to buy my daughter’s gas because they didn’t really have the means for her to bring me [to UAB]. And my daughter would have to sacrifice, most of the time, her job till… I didn’t realize it was becoming a problem. I didn’t have to, but I’ve even paid them something for taking me, because they’re taking off from their job.
		23	For babysitting. That was pretty much 250 every two weeks, depending on who I get basically to come to the house and sit with them, because they couldn’t come up [to UAB]. None of the kids can come up there…I started having Care.com, one of their babysitters to come out. So whenever I had treatment, they’ll come out and sit. And yeah, they charge a nice penny, but it was well worth it.
	Medical costs	14	And even though I have primary insurance and secondary insurance, I know from previous experience those two do not cover everything that your doctor recommends. And especially with something like me having Stage 3 triple-negative breast cancer, [the reimbursement] is a really big deal, knowing that you have some backup if your insurance says, “No, we’re not going to cover this,” even though the doctor feels like this is going to affect your entire life.
		31	I prepared myself, I bought a wig, I bought caps because it may come to that I will lose my hair. But it’s so much more than just hair. And it’s not that I’m vain or it is just so much more, it’s a reflection of you in the mirror that you don’t recognize the person that you see. And when I wake up in the morning and I see my head with hair, and I’m like, “Oh, I feel good today.” And it’s just quality of life for the patient for sure.
	Home-related	12	I was real worried about the money situation. I mean, I’ve got good insurance, but still there’s other things sometimes that insurance might not cover and stuff. But [the reimbursement] helped me because I had some other bills that I…Well, it was a big water bill where I had a leak and couldn’t get it fixed right off the bat. And that helped me out. I got to keep water.
Impact of reimbursement on trial recruitment and retention	Noncoercive nature of reimbursement	14	While I’m grateful and it’s fantastic, and I’m so happy to have that cushion during those times, it’s not appealing. It’s not like, “Oh, let me do this research so I can get money.”
	Enrolled due to impact on prognosis	14	[The reimbursement] would absolutely not affect my decision of wanting to give myself the best chance of care and the best opportunity to live the longest life that I can. I’d be like reimbursement wouldn’t be in the realm of things I would need to decide for my care.
		40	It was definitely based on getting the best treatment for myself…it would not have impacted my decision to start the clinical trial. It was just a bonus… If it’s something that gives me more time here to be with my child, that’s all that matters to me.
	Enrolled due to receiving cutting-edge therapy	7	It just was a nice added bonus to me…The reason why I wanted to participate in the trial is because I figured that it’s at the cutting edge of what is available to help me. And there would be more done as far as my breast cancer goes in a clinical trial, then there would be the typical treatment.
	Enrolled due to increased monitoring by health-care team	25	I was iffy about the trial…[but] I liked the attention. I liked it that you guys, the trial was more on hand. I saw my oncologist more…I think that the financial part of it was definitely a plus. It was helpful. It was.
	Enrolled due to trust in oncologist opinion	20	I was so thankful for this opportunity because Dr [oncologist], I mean I value her opinion and what she thinks should be my next step, and I was just so thankful because she said that she thought I would be a good fit for it so basically cost wouldn’t have really… that wouldn’t have affected my decision.
	Enrolled due to altruism	30	As one nurse told me…the drugs, the chemo that was out there prior to what you’re taking now was a clinical trial. So every drug that you are taking has been a clinical trial drug. And honestly, and one of my decisions was, well, if I can take these drugs and it works, maybe I can help other women down the road to be able to get it. And it won’t be a trial anymore…You can’t put a price on your health is what I have found.
	Aided with retention	7	I would have enrolled regardless…[but] knowing that even if it was difficult to find child care, it wasn’t going to place a financial strain on us because of these reimbursements, that definitely made it less stressful… It made it easier for me to feel good about continuing with the trial.
Impact of reimbursement on financial hardship	Psychological financial hardship	13	[The reimbursement] made me feel like I was in control of what I was doing…it kept me from having to worry about what I had to focus on, that I could just sit back and relax and see how I was feeling.
		17	I think when you start looking at the personal situations of whether the stipend is beneficial to someone and finding out what their needs are, for me, it came down to I’ve been able to provide my own food and have a place to live and to reimburse the ones that are taking me back and forth to the hospital with that money. That’s been a life-saving gift for my dignity, I’m not having to burden someone.
Suggested modifications to the reimbursement intervention	Tailored reimbursement amount	8	I can’t even put an exact dollar amount on it because everybody’s situation is going to be different. I will say that that amount helped me because anything more than what I had at the moment helped me. I’m very grateful for anything, but somebody that’s in a different situation…somebody else might need more than that.
		45	I can see where for a lot of people, they might need some more depending on where a lot of different factors, where you live, how far you have to drive, what shape your car is in if you’ve got a ride, that type thing. But for me, it was good and covered just about everything I needed.
	Lengthened reimbursement duration	16	I’m very appreciative of the reimbursement and it was plenty. I have no complaints about it, no more than just, even if the funds had to be cut from the beginning just to stretch it out a little longer while we still in the trial.
		39	Why is it so short? We’re on this trial for months, almost a year. It was only four months, and a lot of us are not working. So that money really came in handy as far as helping out with bills instead of trying to find resources.

#### Trial-related expenses incurred

Almost all patients (96%) reported using the financial reimbursement to pay for trial-related food (89% groceries, 61% restaurants while at trial visits, 32% specialty foods due to receiving treatment), stating, “There were a lot of times where … I was not feeling up to cooking dinner, not having time to cook dinner. So the expenses, as far as having to pick up food from restaurants, that was an expense that we don’t normally incur quite to that level (P10).” The majority of patients (89%) also used the reimbursement to cover trial-related transportation expenses (75% gas, 29% parking, 11% hotel, 11% car maintenance), noting, “Every time I go back and forth, that’s probably $60 in gas … because of some of the tests that I had, I had to come there back and forth, sometimes two days in a row. So instead of driving back, I got a room (P8).” Most patients (68%) also used the reimbursement for caregiver expenses (43% caregiver support, 25% child care), stating, “[I reimbursed] my friends and family for gas, their meals when they bring me, things that you just don’t think, ‘Okay, somebody’s sitting in chemo with me for eight hours. I need to buy them a sandwich on the way out (P14).’” Over half of patients (64%) used the reimbursement for out-of-pocket medical costs (50% cost-sharing, 29% prescriptions, 21% supportive care), noting, “I paid copays every time I got [the trial drug]. Even though insurance is covering it, you’re still out of pocket a certain amount, and that’s not counting [supportive treatments like] when you catch mouth sores, that’s $90-something mouthwash, my insurance didn’t cover it (P25).” Finally, 54% of patients used the reimbursement for home-related costs (43% utilities, 11% cleaning, 11% rent/mortgage), with one saying, “[The reimbursement] helped me because I had … a big water bill where I had a leak and couldn’t get it fixed right off the bat. And that helped me out. I got to keep water (P12).”

#### Impact on trial recruitment and retention

Patients (97%) did not feel coerced to enroll in the clinical trial due to the reimbursement, stating the reimbursement was a “bonus” or a “blessing.” One patient said, “It wasn’t about the money. The trial was to find out if there’s a different route, a different cure to help somebody else to get better, rather than worrying about $1000 or $5000 being sent to you (P23).’” Instead, patients described enrolling in the clinical trial due to a myriad of reasons, including its impact on their prognosis (27%), with one patient saying, “I was going to do it anyway. If I had to beg, plead, go outside and hold out a cup, whatever I had to do, I was going to do it because I wanted to be alive (P8).” Patients also enrolled due to receiving cutting-edge therapy (33%), increased monitoring by the health-care team (9%), and based on their oncologist opinion (12%), noting, “[The clinical trial nurse] did say there would be a lot more tests, but the way I looked at that, they were following me very closely. So I thought that was a good thing. So even though we have to make the long trips, it’s just part of getting the best care is the way I look at that. But there’s extra expenses for sure (P43).” Patients also mentioned altruistic motivations for participation (15%), stating, “I mainly do this so in the future cancer treatment like I-SPY is the future for patients and give them quality of life. And I feel honored to participate in it knowing that my numbers are helping one way or the other (P31).”

Although the reimbursement did not affect trial recruitment, patients reported the reimbursement aiding in their trial retention (70%), with one saying, “I would have enrolled regardless … [but] knowing that even if it was difficult to find child care, it wasn’t going to place a financial strain on us because of these reimbursements, that definitely made it less stressful. … It made it easier for me to feel good about continuing with the trial (P7).” Another stated, “Just knowing that I could get the reimbursement was a help, because I didn’t know what to expect and what was going to be coming … even just helping out with something to eat while I was waiting [at clinic] because I would be there all day (P28).” Still another patient said, “The boost I had from [the reimbursement], it did help out for me to continue [the trial]…and with me having to take off [work] so much and go through the things that I was going through, being supported financially was a plus (P25).” Finally, “It took maybe, three or four trips [to UAB]. … I was getting broke already. It really subtracted from me very fast. I didn’t expect it to do that. I was racking up credit card debt, I was putting things on Afterpay. I knew it was going to catch up to me, so the fact that [the reimbursement] was there really helped me [to continue participating in the clinical trial] (P6).”

#### Impact on financial hardship

The reimbursement affected patients’ experiences of trial-related financial hardship materially by aiding in addressing trial-related expenses, behaviorally through helping patients stay on trial, as well as psychologically. One patient said, “[Regarding] whether the stipend is beneficial. … I’ve been able to provide my own food and have a place to live and to reimburse the ones that are taking me back and forth to the hospital with that money. That’s been a life-saving gift for my dignity, I’m not having to burden someone (P17).”

#### Suggested modifications

Patients suggested 2 modifications to the financial reimbursement intervention. First, 45% of patients recommended tailoring the reimbursement amount to individual needs, stating, “Some people may need more. Some people may need less. I think it just really depends on the individual. … I sure would hate for someone to miss out on a clinical trial or being treated, just because they can’t pay what the clinical trial doesn’t pay for (P22).” Second, 12% of patients recommended lengthening the reimbursement duration, saying, “I just think they should continue that reimbursement to the end of the trial. … [I’m] going back to work, I got to build money back up for my bills (P38).”

## Discussion

This pilot study found financial reimbursement for clinical trial-related costs was feasible in a sample of therapeutic clinical trial-eligible women with breast cancer at a large, academic medical center located in the US Deep South. Furthermore, all patients in our study remained on their parent clinical trial, suggesting that financial reimbursement may improve trial retention, as well as preserve medical behaviors such as adherence to recommended medical tests, specialists, or follow-up appointments that may be skipped due to trial-related costs. Patients reported feeling less stressed about continuing on the parent clinical trials due to the financial support provided by the reimbursement to cover unforeseen expenses related to their trial participation, such as lost wages, child care, and food while attending long clinic days. Our study results support and align with other impactful research-based efforts to provide financial compensation and increase participation and equity within cancer clinical trials.^24^^,^^25^^,^^38^^,^^49^^,^^50^ However, our study differs from these previous efforts because our participants were not required to submit claims for reimbursement via receipts, allowing for greater flexibility in use of the money. This allowed patients to pay for items that are difficult to quantify or often denied reimbursement, such as productivity losses, caregiver expenses, or supportive treatments or devices that insurers may not consider medically necessary. Our study also adds to the literature by identifying the types of costs patients are incurring while participating in a clinical trial. Overall, our results support further studies on financial reimbursements as tools to increase cancer clinical trial participation, diversity, and increased generalizability of research findings.

A primary criticism of financial reimbursement for trial-related expenses is the potential for undue influence or coercion to participate in research. However, patients in our study emphasized their participation was motivated by factors other than the reimbursement, such as the impact of the trial on their prognosis, ability to receive new and cutting-edge therapy, increased monitoring and surveillance by their health-care team, trust in their oncologist’s opinion, and altruism for future patients with cancer. This finding aligns with other research, where clinical trial participants believed monetary reimbursement would not coerce individuals to participate if they found the trial protocol unacceptable at enrollment, and would instead act as beneficial compensation reflecting the time, inconvenience, and risks related to participation.^51^ Notably, the Food and Drug Administration (FDA) considers payment for research participation a “just and fair” research practice if it does not interfere with patients’ ability to give voluntary informed consent.^52^ Similarly, the American Society for Clinical Oncology issued a policy statement in 2018 that advocated for patient protection from trial-related financial expenses “as a matter of justice,” because they are crucial contributors to scientific and therapeutic advancement.^53^ Therefore, many researchers and bioethicists have concluded restricting clinical trial participation based on a patient’s ability to pay is inherently unethical due to impeding their ability to receive a potentially life-saving treatment, as well as hindering the speed and generalizability of research.^26^^,^^29^ Rather, the ethical course is provision of financial reimbursement high enough to protect against patient exploitation or overburden during trial participation, insuring treatment access, appropriateness, and increased quality of life via access to basic needs, such as food, for patients with cancer.

Although this pilot study was not powered to show statistical significance, we saw modest decreases in patient financial toxicity over the 4 months that patients were enrolled in our reimbursement study, potentially due to reimbursements helping patients cover trial-related expenses related to food, transportation, caregiving, medical expenses, and home-related costs. These small decreases were similar to those reported by Nipp and colleagues within a cancer clinical trial equity intervention.^50^ Notably, Black patients in our study started with higher levels of financial hardship and reported greater decreases in financial hardship compared with White patients, suggesting potential for financial reimbursements to increase trial sample diversity by reducing the trial’s impact on financial hardship for patients with higher financial hardship at baseline, such as Black patients. Although previous research has suggested strategies such as better integration of community engagement during trial design, conduct, and dissemination to build patient trust or decentralization of trials to promote patient accessibility,^54^ our study addresses a key barrier to diverse trial participation, because Black patients in our study reported greater financial hardship than White patients upon enrollment. Future research testing these strategies in combination is needed to identify the most optimal way to allow increased diversity in clinical trial participation.

Patients in our study also suggested modifications to the reimbursement intervention that could result in even greater reductions in financial toxicity, such as tailoring the reimbursement amount to individual participant financial situations, as well as lengthening the duration of reimbursement. These modifications are endorsed by the FDA, because it encourages institutional review boards to consider patient time, inconvenience, and discomfort in calculating reimbursements for clinical trials. Furthermore, because clinical trials can last from many months to many years, thus putting patients at risk of financial toxicity for long periods of time, the FDA also recommends remitting payments to participants as the study progresses rather than at study completion alone.^52^ Future iterations of trials testing the impact of financial reimbursement for clinical trial participation should consider incorporation of these suggestions for greater patient centeredness of the trial, potentially leading to greater improvements in trial retention.

The financial reimbursement in our study was supported by philanthropic foundation–based research funding. However, the sustainability of this funding mechanism to reimburse trial-related costs is questionable. Thus, if financial reimbursements or stipends for research participation were to be implemented as standard care, who bears the responsibility of paying? Federal sponsors, such as the NIH, allow investigators to budget patient costs for research participation, such as transportation, meals, child care, or financial incentives, into their funding.^55^ However, investigators supported by federal research funding often deal with extremely conservative paylines and tight budgets that may not allow for the increasingly high costs of trial participation to be adequately addressed. Additionally, recent pushes to constrain the NIH budget may further restrict the amount of federal funding available for these budget items to adequately reimburse participants, or potentially risk the ability to even conduct clinical trials. For industry sponsors, who enroll cancer clinical trial participants on an 8:1 ratio compared with federal sponsors,^56^ legal barriers such as Anti-Kickback Statutes may currently impede payments to participants for trial-related expenses. However, the May 2025 Clinical Trial Modernization Act (H.R. 3521)^57^ seeks to provide safe harbors for trial sponsors to provide compensation to trial participants. Financial reimbursement may also be a cost-effective method for retaining clinical trial participants and reducing profit losses from trial attrition and delays in patient enrollment. A study by Moore and colleagues estimated a sponsor spends $41 117 per patient enrolled on trial, $3562 per patient trial visit, and a cost difference of $31M for studies open greater vs <26 weeks.^58^ Based on this pilot data, research testing the efficacy and cost-effectiveness of financial reimbursement is needed to understand the impact on the responsible payor.

Our study finding should be considered within the context of limitations. This was a small, single-site pilot study of only women with breast cancer. Our results may not be generalizable to other academic medical centers or cancer types. Future research testing the efficacy and effectiveness of financial reimbursement for cancer clinical trial expenses is therefore warranted.

## Conclusion

Provision of financial reimbursement for clinical trial–related costs was feasible, acceptable, and appropriate in a Deep South sample of therapeutic clinical trial-eligible women with breast cancer. Findings suggest receipt of financial reimbursement may improve trial retention, may not be coercive to trial recruitment, and may decrease trial-related financial toxicity. Our results indicate financial reimbursement as a promising method to increase both cancer clinical trial retention and diversity, warranting further investigation to be established as an important tool to optimize the pace of scientific research and generalizability of research findings.

## Supplementary Material

pkag048_Supplementary_Data

## Data Availability

The data that support the findings of this study are available on request from the corresponding author. The data are not publicly available due to privacy or ethical restrictions.

## Prior presentations

Results from this study were selected for an oral abstract presentation at the 2025 ASCO Quality Care Symposium.
