# Supplementary material for: Financial reimbursement for clinical trial participation costs: a pilot feasibility study
Source: JNCI Cancer Spectr. 2026 May 3;10(3):pkag048. doi: 10.1093/jncics/pkag048 (PMC13228128; doi:10.1093/jncics/pkag048)
Supplement: pkag048_Supplementary_Data [file pkag048_supplementary_data.docx]

Table S1. Reimbursement “dose” de-escalation criteria.

| **Reimbursement Dose Suitability Criteria** | **Instrument** | **Scoring** | **De-Escalation Criteria** |
| --- | --- | --- | --- |
| Negative financial toxicity screen | COmprehensive Score for financial Toxicity (COST) | Scored 0-44, scores < 26 indicate financial toxicity | Average  score ≥ 26 |
| Reimbursement dose acceptable | Acceptability of Intervention Measure (AIM) | 4-item, 5-point Likert scale; higher scores indicate greater acceptability | Average  score ≥ 4 |
| Reimbursement dose appropriate | Intervention Appropriateness Measure (IAM) | 4-item, 5-point Likert scale; higher scores indicate greater appropriateness | Average  score ≥ 4 |

Figure S1. Study schema.


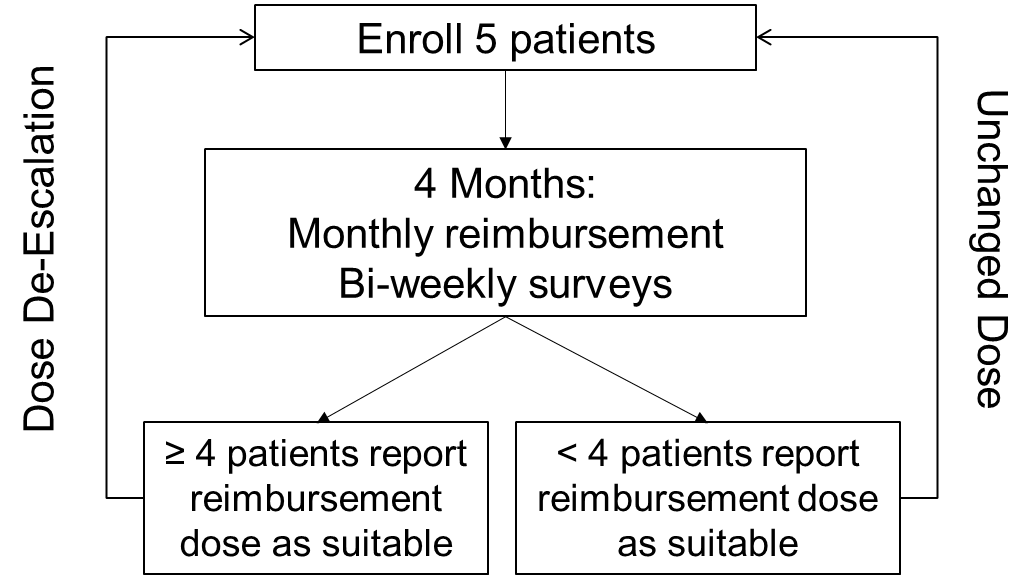


Figure S2. Semi-structured interview guide.

Introduction

Good [morning/afternoon/evening]. My name is [name] and I’m a [job] at UAB. This interview is a part of a study looking at the financial impact of cancer care and ways that we can help support patients who are participating in clinical trials. As you’re currently enrolled in a clinical trial and are receiving monthly reimbursements for your participation, we want to talk to you about what your experience has been like and how, or even if, those reimbursements have been helpful to you.

The interview should last around 30 minutes. All of my questions will be open-ended and focused on your experience, so there really are no right or wrong ways to respond. Please feel free to be as candid as you’re comfortable with, because we genuinely do want to help support people as best as we can. Anything you say to me today will remain anonymous and confidential, and it won’t have any impact on the care you receive at UAB.

If it’s okay, I would also like to record this interview, so that I can focus on our conversation now, but can also go back and compare your responses to others that I speak with later on. Are you okay with me recording? [START RECORDER]

Do you have any questions before we begin?

(*If no questions or after questions are resolved):* Okay, if you have any questions as we go, please feel free to stop and ask me at any time.

### Interview Questions

1. Let’s start by talking a little bit about your experience getting diagnosed with breast cancer. How did you first realize that something was wrong?
   1. And, how long have you been participating in the clinical trial?
   2. What cancer care have you received as part of the trial? (*Probes:* chemotherapy, MRIs, biopsies, blood draws, genetic testing)
2. Is this the first clinical trial that you’ve participated in?
   1. IF YES
      1. How many other clinical trials had you participated in?
      2. Do you feel like you were financially impacted by your previous participation in trials?
   2. IF NO
      1. Have you been approached to join other trials?
         1. IF YES, do you remember why you ultimately decided not to participate in them?
      2. Prior to participating in this study, had you ever thought about the financial impact of being in a clinical trial?
3. When you were deciding to participate in a clinical trial, did you have any discussions with anyone on your care team about the financial costs of participating?
   1. *If discussed:*
      1. Who did you discuss costs with?
      2. What sorts of things did you talk about?
      3. Who would you have liked to have discussed costs with?
      4. Did anything that you talked about with them influence your treatment decision? What?
      5. Were your family or friends ever involved in the discussion? If not, would that have been helpful?
   2. *If not discussed:*
      1. What cost-related information would you like to have known before participating and why?
      2. Who would you like to have discussed costs with?
4. Now let’s talk a little about what your participation in the clinical trial has been like.
   1. How much time per week would you say you spend doing trial-related things?
      1. (*Probes:* visiting your doctor, receiving infusions, visiting clinic for labs/scans)
   2. And how often and far do you have to travel to UAB each time?
      1. So, you would say that you usually spend how much time travelling each week?
5. What have been the main financial expenses associated with your participation in the clinical trial?
   1. How much would you say that you were you spending out-of-pocket to participate each week?
      1. *Probes:* trial-related labs, imaging, procedures, out-of-network care, fuel, parking, rental car/ride share, hotels, food, lost wages
   2. What trial-related costs did the trial sponsor cover/not cover?
   3. What trial costs did your insurance cover/not cover?
   4. Were there any costs or financial concerns that you encountered during the trial that you didn’t expect beforehand?
   5. Outside of the monthly reimbursements, did you receive any other financial assistance for your cancer care?
6. What sorts of things have you used the monthly reimbursement money to cover while you’ve been on trial?
   1. Do you feel like the reimbursement amount was enough to cover all costs of participating in a clinical trial?
   2. Were there any trial-related expenses that it didn’t cover?
   3. Are you still experiencing any financial burdens related to the clinical trial now that you’re receiving monthly reimbursements?
   4. What do you think is a fair and useful monthly reimbursement amount for clinical trial participants and why?
7. Outside of your cancer treatment, have the monthly reimbursements made any impact on how you feel about your personal finances?
8. To what extent would you say that the monthly reimbursements have affected your decision to enroll and then continue your participation in the clinical trial?
9. How would receiving full reimbursement for trial-related participation costs affect your willingness to participate in future clinical trials?
   1. Based on your trial out-of-pocket expenses, what reimbursement amount would be fair for trial participants? Why?
   2. In your opinion and based on your experience, what should researchers consider when deciding how much to reimburse study participants?
10. Is there anything else you would like to add before we go?

Thank you very much for taking the time to participate in this study.
